# A revised application of cognitive presence automatic classifiers for MOOCs: a new set of indicators revealed?

**DOI:** 10.1186/s41239-022-00353-7

**Published:** 2022-09-13

**Authors:** Yuanyuan Hu, Claire Donald, Nasser Giacaman

**Affiliations:** Faculty of Engineering, The University of Auckland, Auckland, New Zealand

**Keywords:** Cognitive presence, MOOC, Online discussion, Feature importance, Automatic text analysis

## Abstract

Automatic analysis of the myriad discussion messages in large online courses can support effective educator-learner interaction at scale. Robust classifiers are an essential foundation for the use of automatic analysis of cognitive presence in practice. This study reports on the application of a revised machine learning approach, which was originally developed from traditional, small-scale, for-credit, online courses, to automatically identify the phases of cognitive presence in the discussions from a Philosophy Massive Open Online Course (MOOC). The classifier performed slightly better on the MOOC discussions than similar previous studies have found. A new set of indicators to identify cognitive presence was revealed in the MOOC discussions, unlike those in the traditional courses. This study also cross-validated the classifier using MOOC discussion data from three other disciplines: Medicine, Education, and Humanities. Our results suggest that the cognitive classifier trained using MOOC data in only one discipline cannot yet be applied to other disciplines with sufficient accuracy.

## Introduction

In this section, we introduce one of the main problems that educators, learners, and researchers encounter in Massive open online courses (MOOCs) teaching and learning. Then, we clarify the significance of the study, and research purpose and research questions in relation to the problem.

### The problem

Massive open online courses (MOOCs), which originated from the Open Educational Resources movement, have attracted much public attention, especially during the COVID-19 pandemic (Buchem et al., 2020; Lohr, 2020). MOOCs differ from the traditional brick-and-mortar university courses in terms of pedagogical design, open and free access to the learning content, a more diverse range of learner profiles, and a variety of learning objectives (Alario-Hoyos et al., 2017; Gardner et al., 2018). These differences raise significant challenges for the MOOC providers and designers due to the scale and heterogeneity of learners. These challenges included providing efficient and effective feedback from the instructors, a collaborative learning environment, and flexibility of the personal learning schedules (Zhu et al., 2018). A major challenge is that with the limited number of MOOC educators it is practically impossible to monitor the learning progress of millions of MOOC learners and provide them nuanced and accurate guidance individually (Almatrafi et al., 2018; Galikyan et al., 2021). MOOC learners also need responsive and high-quality feedback to guide their self-paced learning engagement (Hu et al., 2021). The lack of educators’ attention and responses becomes obstacles for the learners to move forward (Almatrafi et al., 2018).

Asynchronous online discussion forums play a key role in assisting the participants’ mutual interaction through textual conversations in MOOCs. These discussion transcripts can provide instructors with an understanding of learners’ critical discourse (i.e., knowledge construction) during the course. The Community of Inquiry (CoI) framework (Garrison et al., 1999) has been the most broadly used and validated for analysing educational experience in online discussions. Cognitive presence, a primary dimension of the CoI, focuses on the critical discourse during learning. The cognitive presence reflected in the discussion messages is significant evidence that students are learning domain knowledge (Rourke & Kanuka, 2009). Using such a framework to categorise learners’ discourse in online discussions is a formidable task that could be very helpful for educators to monitor students’ learning progress (Elgort et al., 2018). It is also not practical to implement the manual categorisation process in the myriad discussions to assist teaching and learning at scale (Kovanovic et al., 2014). A reliable and high-performing approach to automatically analysing cognitive presence in MOOC discussions can be applied in MOOC platforms as an effective tool to enhance the communication between the relatively small number of educators and the vast number of learners. This offers a potential solution to one of the major challenges of MOOCs.

### The significance

Towards the goal of implementing the automatic classifiers into practical use in MOOC discussion forums, the automatic content analysis methods developed for the discussion transcripts in the small-scale courses need to be revalidated for the MOOCs. The informal writing styles are distinctive in the MOOC discussion forums compared to the formal writing patterns in the for-credit courses (Hu et al., 2021). These specific linguistic patterns can impact the architecture and feature extraction in natural language processing methods for identifying the phases of cognitive presence. Some automated and semi-automated approaches for analysing cognitive engagement have been proposed in the past two decades (Barbosa et al., 2020; Corich et al., 2004; Farrow et al., 2019; Kovanović et al., 2014, 2016; McKlin et al., 2001; Waters et al., 2015), amongst which Kovanović et al.’s (2016) classifier reached the state-of-the-art performance. However, these studies all concentrated on the context of the traditional, for-credit, small-scale university courses (called the small-scale courses for short) rather than MOOCs. Also, these previous studies worked on the discussion messages from different disciplines. The generalisability of the classifiers constructed by the data sets from one domain to another requires to be validated, too. Besides, the explainable machine learning methods (e.g., random forest) can help researchers seek important indicators for each cognitive presence phase. Researchers can refine the theoretical frameworks (i.e., the CoI) for their generalisability and transferability in broader disciplines and learning environments based on these important indicators.

### Purpose and the research questions

This study aims to examine and revise Kovanović et al.’s (2016) classifier to identify cognitive presence phases in the discussion messages from a target MOOC. Kovanović et al.’s (2016) classifier applied the random forest algorithm with the features based on discussion structures (e.g., the depth of conversation), psychological vocabularies (e.g., “think”), and cohesion analysis of writing texts (e.g., the semantic relevance between two sentences in a message). The most important features to identify the phases of cognitive presence were analysed to gain insights for further studies of cognitive presence in MOOCs. The best-case classifier was also validated on the sample messages of MOOCs from the other disciplines. Thus, our research questions were:

RQ1: To what extent can our automatic classifier accurately identify the phases of cognitive presence in the online discussion messages from the target MOOC?

RQ2: Which classification features can be the most important to identify each phase of cognitive presence according to the automatic classifier training results?

RQ3: Can the automatic classifier trained on the target MOOC potentially identify cognitive presence in MOOCs of the other disciplines?

## Related studies

We introduce some theoretical background about the Community of Inquiry (CoI) framework and one of its core dimensions, cognitive presence, in this section. The prior studies on the automated classifiers of cognitive presence in online discussion transcripts are elaborated after the theories. Also, the gaps between this research and the previous studies are explained at the end of the section.

### The Community of Inquiry (CoI) framework and cognitive presence

The CoI framework proposed by Garrison et al. (1999) has been most broadly cited for analysing learning in asynchronous online discussion forums in the past two decades. Based on the theory of social constructivism, the CoI portrays the educational experience occurring in a learning community where “a group of individuals who collaboratively engage in purposeful critical discourse and reflection to construct personal meaning and confirm mutual understanding” (Garrison & Anderson, 2011, p. 2). The CoI framework is composed of three interdependent elements, also called presences: (1) Cognitive presence, denotes the progressive phases of knowledge (re)construction and problem-solving skills (Akyol & Garrison, 2011); (2) Social presence, describes the development of social climate and interpersonal relationships between the participants in the learning community (Rourke et al., 1999); (3) Teaching presence, reflects the instructional activities that facilitate and intervene in the construction of critical discourse (Garrison et al., 1999).

Since our study concentrates on the construction and facilitation of ‘critical inquiry’ in text-based discussion forums in MOOCs, we adopted the definition of cognitive presence in the CoI framework. Cognitive presence, which is represented as a cycle of progressive knowledge construction (Garrison et al., 2001), has four phases: (1) Triggering event, in which participants raised their confusions and were unable to explain the causes explicitly; (2) Exploration, in which the participants provide information to explore the triggers of the confusions in the previous phase; (3) Integration, in which the participators proposed coherent conclusions or solutions to address the confusions mentioned with sufficient supporting reasons; (4) Resolution, in which the participators applied, tested or argued the conclusions or solutions mentioned in the former phases, forming new constructs. The discussion messages associated with the other two presences (i.e., social and teaching presence) and do not belong to any of the above four phases are classified into the Other. According to the coding-up rule, the discussion messages that reflect the evidence of more than one cognitive phase are categorised into the higher one. Table 9 in the Appendix provides message instances of the five categories associated with the definitions in cognitive presence.

### Automated classifiers of cognitive presence in online discussion transcripts

Researchers have developed several automated classifiers using different algorithms to analyse the phases of cognitive presence in online discussion transcripts of the small-scale courses. Table 1 summarises the methods, main features, and outcome metrics of the studies reviewed in this section.

**Table 1:** Summary of prior work reviewed

Studies by	Algorithm	Main features	Best outcome metrics	
			Accuracy (%)	Cohen’s κ
McKlin et al. (2001)	Simple neural networks	Dictionary-based words and phrases	68	0.31
Corich et al. (2006)	Bayesian network	Dictionary-based words and phrases	71	–
Kovanović et al. (2014)	Support vector machine	Bag-of-words, n-grams, and structural features	58.4	0.41
Waters et al. (2015)	Conditional random fields	Bag-of-words, n-grams, and more structural features	64.2	0.48
Kovanović et al. (2016)	Random forest	LIWC, Coh-Metrix, LSA, structural features	70.3	0.63
Neto et al. (2018)	Random forest	LIWC, Coh-Metrix, word embeddings, structural features	83	0.72
Farrow et al. (2019)	Random forest	Same as Kovanović et al. (2016)	61.7	0.46
Barbosa et al. (2020)	Random forest	Same as Kovanović et al. (2016)	67	0.32
Neto et al. (2021)	Random forest	Same as Kovanović et al. (2016)	76 67^a^ 57^b^	0.55 0.2 0.38

^a,b^Neto et al. (2021) contains three experiments. The first one was on a combined data set. The next two were training the automatic classifier on one set and testing on another, and vice versa

Initially, McKlin et al. (2001) and Corich et al. (2006) applied a simple artificial neural network (ANN) and a Bayesian network, respectively, by using dictionary-based words and phrases as classification features to categorise the cognitive presence phases. McKlin et al.’s (2001) ANN classifier reported Holsti’s coefficient of reliability (CR) of 0.68 and Cohen’s κ of 0.31, while Corich et al.’s (2006) classifier reached the CR of 0.71 but without any report of Cohen’s κ. These results indicated that there was still much room for optimisation. Also, McKlin et al.’s (2001) classifier excluded the minority class, Resolution phase. Corich et al.’s (2006) classifier analysed cognitive presence based on sentence level rather than message level.

Subsequently, Kovanović et al. (2014) built a cognitive presence classifier by Support-Vector-Machine algorithm with n-grams and thread structures as classification features. Kovanović et al.’s (2014) classifier archived an accuracy of 58.4% and Cohen’s κ of 0.41. By adding more structural features, Waters et al. (2015) developed a Conditional random fields classifier. It reached an accuracy of 64.2% and Cohen’s κ of 0.482, demonstrating the importance of the structural features for identifying cognitive presence phases in online discussions.

However, the limitations of the n-grams methods were (1) they built a high-dimensional space that caused over-fitting problems, and (2) they led the classifier domain-specifically so that it lacked generalisability. Also, the skewed distribution of phases of cognitive presence in the sample data was another problem that affected the classifier performance. To overcome these problems, Kovanović et al. (2016), as the state-of-the-art method, built a random forest (RF) classifier with the features based on computational linguistics analysis by Coh-Metrix (Graesser et al., 2014) and Linguistic Inquiry Word Count (LIWC, Tausczik & Pennebaker, 2009), latent semantic analysis (LSA), name entities, and conversational structures. It also employed over-sampling techniques to address the class imbalance problem. It reached an accuracy of 70.3% and Cohen’s κ of 0.63. However, the accuracy and Cohen’s κ in a replication study by Farrow et al. (2019) decreased to 61.7% and 0.46, pinpointing that Kovanović et al.’s (2016) approach generated an over-optimistic result since it performed the over-sampling method before the training-test data split.

Several revised random forests approaches were also applied in cross-language studies (Barbosa et al., 2020; Neto et al., 2018) which aimed to categorise the phases of cognitive presence in the discussion messages written in Portuguese. Neto et al.’s (2018) study reported an accuracy of 83% and Cohen’s κ of 0.72, whereas Barbosa et al.’s (2020) work reached a lower performance, with an accuracy of 67% and Cohen’s κ of 0.32. Neto et al. (2021) used the revised approach to classify the phases of cognitive presence in the discussion messages combined two discipline courses, biology and technology, achieving an accuracy of 76% and Cohen’s κ of 0.55. They also evaluated the performance of the automatic model trained by the messages from one discipline on another discipline, with the results of Cohen’s κ below 0.4, indicating the approach was not generic enough.

### Differences between the current and the prior work

There were three main differences between the current and the prior work. Firstly, the studies that analysed the learners’ cognitive aspects in the MOOC discussions focused on cognitive engagement behaviours, sentiment analysis, learners’ confusions rather than the phases of cognitive presence. Secondly, research in the literature that analysed cognitive presence manually or automatically was in the context of small-scale courses rather than MOOCs. Finally, the domains of the courses investigated by the reviewed studies were different, for instance, political and history (McKlin et al., 2001), software engineering (Farrow et al., 2019; Kovanović et al., 2014, 2016; Waters et al., 2015), biology (Barbosa et al., 2020; Neto et al., 2018, 2021). It is still a doubt whether the automatic classifier that was developed for a specific MOOC course can be applied to the MOOCs from other disciplines. The methods are discussed below to fulfil the three differences mentioned above.

## Methods

We ran a revised application study to address the research questions introduced at the end of the “Introduction” section. We first describe the data sets used in the study. We then elaborate on the construction of the automatic classifiers by introducing the classification features used to identify phases of cognitive presence, model training and testing processes, and validation of the optimal classifiers on the discussion messages of other disciplines.

### Description of the data sets

In this study, the data set used to build the automatic classifier for phases of cognitive presence came from an archived offering of the Logical and Critical Thinking (LCT) MOOC. This introductory Philosophy MOOC was designed and taught by a course-design team at a New Zealand university on the FutureLearn platform. This course taught the basic concepts of logical and critical thinking and how to build sound arguments linking with daily life. The philosophy MOOC sample data was composed of 1917 discussion messages (including threads and their comments) that we randomly selected from 12,311 messages generated by 1000 learners in the forums from eight weekly topics. Two tasks were randomly selected from each week’s topic, and then approximately 100 messages were randomly selected from each task to keep the sequential structure of a thread. Three expert coders classified the 1917 messages into five phases of cognitive presence (77.15% agreement, Fleiss’ κ of 0.763) independently based on a revalidated classification rubric for cognitive presence in MOOC (Hu et al., 2020). The messages categorised in the same phase by all the three coders (1479 messages) were used to develop the automatic classifier in this study. Table 2 shows the proportion of messages within the five phases of cognitive presence in the Philosophy MOOC data set.

**Table 2:** Distribution of cognitive presence phases in the sample data from the MOOCs of Philosophy, Medicine, Education, and Humanities

Id	Cognitive phase	Philosophy set		Medicine set		Education set		Humanities set	
		Count	%	Count	%	Count	%	Count	%
0	Other	85	5.75	3	3.03	3	3.09	12	12.2
1	Triggering event	279	18.86	36	36.4	34	35.1	27	27.6
2	Exploration	835	56.46	43	43.4	44	45.4	41	41.8
3	Integration	244	16.50	16	16.2	10	10.3	16	16.3
4	Resolution	36	2.43	1	1.01	6	6.19	2	2.04

Count = number of messages

The data sets used for the cross-domain validation of our automatic classifier consist of 307 messages. They were randomly selected from 29,604 discussion messages generated from eleven Stanford University public online courses (Agrawal et al., 2015; Atapattu et al., 2019) in three disciplines (i.e., Education, Medicine and Humanities). Similarly, a sample of approximately 100 messages (i.e., posts and their replies) was selected: 103, 102 and 102 messages for Education, Medicine, and humanity courses, respectively. Two expert coders independently classified the 307 messages into five phases of cognitive presence, according to Hu et al.’s (2020) rubric. They reached an overall percentage agreement of 95.8% and Cohen’s κ of 0.938 (307 messages). Across the three disciplines, Education, Medicine, and Humanities, the percentage agreements were 96.1%, 95.1%, and 96.1%, and Cohen’s κ coefficients were 0.941, 0.926, and 0.945, respectively. Table 3 also lists the distribution of the five cognitive phases in the messages that were categorised in the same phase by both coders.

**Table 3:** Summary of the classifier performance by fine-tuning the parameters (i.e., ntree and mtry)

Fine-tuning process		ntree	mtry	Accuracy (SD)	Cohen’s κ (SD)
With the SMOTE exact method	Min	500	196	0.654 (0.034)	0.414 (0.057)
	Max	**1100**	**54**	0.689 (0.043)	0.465 (0.068)
	Difference			0.035	0.051
Without the SMOTE exact method	Min	500	2	0.659 (0.018)	0.334 (0.040)
	Max	**1100**	**94**	0.694 (0.035)	0.437 (0.069)
	Difference			0.035	0.103

The bold values denote the optimal ntree and mtry values in the fine-tuning processes

All the coders were trained over three rounds to ensure they reached an over 85% agreement before classifying the sample data independently. The distribution of the five cognitive phases accounted for a similar proportion in our four datasets, with a bulk of messages as Exploration phase and a negligible percentage of messages as the Other and Resolution phase. This similarity was also revealed in the datasets of previous studies.

### Feature extraction

Four categories of 225 classification features were adopted for building the automatic classifier in this study, according to the features used and the analysis of their importance for identifying the phases of cognitive presence in the previous studies (Barbosa et al., 2020; Farrow et al., 2019; Kovanović et al., 2016; Neto et al., 2018). The categories contain (1) discussion contextual features, (2) linguistic features, (3) semantic similarities, (4) name-entity words. We briefly explained these features and why we use them as below due to the word limit. Lists of the 225 features and their descriptions can be found in Table 10 in the Appendix.

#### Discussion contextual features

The discussion contextual features have been found to be significant for identifying the cognitive presence phases in the previous studies (Barbosa et al., 2020; Farrow et al., 2020; Kovanović et al., 2016; Waters et al., 2015). Following these studies, four contextual features were used in this study: (1) the message depth, which represents the numeric position (chronological order) within a conversation; (2) the number of replies, which denotes the total number of replies beneath each message; (3, 4) the start or the end message of a thread, which is a binary number (0 or 1) to indicate whether the message is the start or the end of a conversation.

#### Linguistic features

The state-of-the-art studies (Farrow et al., 2019; Kovanović et al., 2016) have found that several classification features from the two computational linguistics tools, Coh-Metrix (Graesser et al., 2014) and LIWC (Tausczik & Pennebaker, 2009), indicated high importance to identify cognitive presence phases. Thus, we extracted linguistic features from these two tools.

The Coh-Metrix offers features to measure the cohesion of texts in five dimensions (Dowell et al., 2016; Graesser et al., 2004): (1) Narrativity measures the degree of using familiar topics or words, world knowledge and oral language to describe events or stories. Narrative text resembles everyday conversation. There is high correspondence with word familiarity. Narrative text would lie at the opposite end of a continuum with less familiar information in expository texts on less familiar topics. (2) Deep cohesion reveals the extent to which the connectives casually or logically help readers to comprehend the ideas expressed in the discourse. (3) Referential cohesion measures the degree to which the explicit ideas are tied together across the entire text. (4) Syntactic simplicity reflects the extent to which sentences contain fewer words and use simpler, familiar syntactic structures to express ideas in the text. (5) Word concreteness evaluates the degree of using concrete and easier words for readers to understand. Texts that evoke meaningful images that are easier to visualise, as opposed to abstract words about concepts that are difficult to visualise and therefore more difficult to understand.

The LIWC tool provides a collection of words as features that indicated various psychological processes, including affective, cognitive, social and perceptual processes (Pennebaker et al., 2015; Tausczik & Pennebaker, 2009). The words that indicate affective processes describe writers’ positive and negative emotions, such as “happy”, or “nervous”. The cognitive process words describe how writers express insight, causation, discrepancy, tentativeness, certainty, and differentiation (i.e., “think” and “consider”). The words related to social processes contain pronouns, nouns and verbs that imply human interactions, such as sharing and talking (i.e., “group” and “collaborate”). The perceptual process vocabulary includes words that suggest perceiving activities such as seeing, hearing, and feeling (i.e., “listen” and “touch”).

#### Semantic similarity

Previous studies found that the semantic similarities of each message with its previous and next message are important indicators for identifying the phases of cognitive presence. The semantic similarity measures how similar or dissimilar the meanings between words, sentences and paragraphs are (Manning & Schütze, 1999, pp. 294–295). We used the most common way to represent the semantic similarity between two messages: the cosine similarity of their term frequency-inverse document frequency (TF-IDF) weighted vectors (Ramos, 2003). The TF-IDF is a very useful way to convert words to numeric vectors, and calculates the number of times each word appears in a collection of documents, but inverts the frequency number. We also used the pre-trained bidirectional encoder representations from transformers (BERT) model (Devlin et al., 2019) as the numeric representations of each message and then computed the cosine similarities between adjacent messages. BERT is a language model developed by Google for pre-training language representations. These obtain bidirectional contextual information by a combining left-to-right and right-to-left training process. It has reported state-of-the-art results in various natural language processing tasks in recent years (Lee et al., 2020; Liu et al., 2019).

#### Name-entity words

State-of-the-art studies report that higher cognitive phases tend to have more name-entity words (e.g., nouns of objects, such as persons, locations, organisations, products). Hence, we extracted 19 name-entity features from the discussion messages using the spaCy library (Honnibal & Montani, 2017), as was done in Neto et al.’s (2018) study.

### Data processing and model training

To address research question 1 and 2, we trained and validated an automatic classifier for the phases of cognitive presence on the sample data from the Philosophy MOOC. We used the 225 classification features with a random forest (RF) algorithm. A RF model consists of a combination of many decision trees to solve regression or classification problems. Each individual tree operates a classification prediction independently and the class with the most distributions in all the trees’ outcomes forms the RF’s results (Breiman, 2001). The sample messages removed numbers and performed lemmatisation and case-folding in the data pre-processing.

#### Optimal parameters

To seek the best-performing RF model, we need to fine-tune two primary parameters, ntree (i.e., the number of decision trees constructed in each training) and mtry (i.e., the number of classification features used by each training tree). For the optimal ntree value, we examined 500 to 1500 sampled with every interval of 200. For the best mtry value, the tenfold cross-validation (CV) method was applied to examine 30 different numbers randomly selected from 1 to 225. The k-fold CV method was applied for minimising over-fitting risks (Casella et al., 2013, p. 181). The entire sample data were randomly split into ten non-repeated folds of the approximately same size (i.e., tenfold CV method). The fine-tuning process was then looped ten times with every nine-fold data as the training set and the remainder as the testing set. We create the final RF classifier with the combination of ntree and mtry value of the best-performing case.

#### The unbalanced class problem

We acknowledge that the skewed distribution of the cognitive presence phases (Table 2) can affect the classification performance. Thus, the SMOTE (Synthetic Minority Over-sampling Technique) exact method suggested by Farrow et al. (2019) was applied in the training process to improve the class imbalance problems. The standard SMOTE method undersamples the majority classes and oversamples the minority classes by generating synthetic data points, which are the nearest neighbouring instances of the existing (original) data points (Chawla et al., 2002). Farrow et al. (2019) extends the standard SMOTE algorithm, which is used to address the binary class problems, to tackle multi-class tasks. Instead of undersampling the majority class, the SMOTE exact method enlarges the number of instances in the minority class into the exact same size of the majority class, which is more appropriate for coping with the limited data in this study. A tenfold CV was adopted to construct the optimal RF classifier with the best ntree and mtry value. The SMOTE exact method was performed in every CV loop to generate more synthetic instances merely in the training folds after the training-test data splits. We also report the performance of the RF classifier without the application of SMOTE exact method as a baseline.

#### Classifier performance metrics

The metrics used to evaluate the performance of the automatic classifiers were accuracy, Cohen’s κ, macro- and weighted-average F_1_ score in this study. The accuracy is the most widely used measure in supervised machine learning tasks. It is defined as the percentage of correctly classified instances over the total number of instances. Cohen’s κ coefficient (Cohen, 1960), which was initially proposed to measure the inter-rater reliability between two human coders, can also evaluate the agreement between the prediction labels and the pre-classified labels. The macro-averaged F_1_ score (Asch, 2013) was used to measure the overall performance across multiple classes, as it regards the classes with fewer instances as equally important as the larger classes. The weighted-averaged F_1_, which has been applied in the text classification tasks in recent years (Chakravarthi et al., 2020), was also adopted to alleviate the impact from the prediction results of the minority class (e.g., the Resolution phase). Moreover, the Mean Decrease Gini (MDG) index, also known as the Mean Decrease Impurity importance, was used to evaluate the importance of classification features for each category. It is a broadly applied measure that adds up the decrease in Gini impurity of each classification feature used for all the nodes in the prediction trees (Louppe et al., 2013).

### Validation of the automatic classifier on the MOOC discussion data sets of other disciplines

To address research question 3, we applied the optimal automatic classifier trained on the Philosophy MOOC set (1,479 messages) to the sample data of other three MOOC data sets: The Education set (97 messages), the Medicine set (99 messages), and Humanities set (98 messages). Same evaluation metrics were used to evaluate the classifier’s performance in the three data sets.

## Results

We demonstrate the results of training and testing our classifiers in the following three subsections associated with the three research questions introduced in the Purpose and the research questions section, respectively. The first subsection clarifies the prediction performance of the classifiers trained and tested on the philosophy MOOC data (*RQ1*). The second subsection reports the important features to predict the phases of cognitive presence in the training processes (*RQ2*). The third subsection displays the validation results that we tested the best-performing classifiers trained by the philosophy data set on the other three MOOC data sets (*RQ3*).

### Model evaluation when training and testing on the Philosophy MOOC data—RQ1

Table 3 demonstrates the performance results, including the accuracy, Cohen’s κ, and the standard deviations (SDs), of the RF classifiers by fine-tuning the two parameters (i.e., ntree and mtry) with and without the application of the SMOTE exact method. These results indicate that the ntree value of 1100 was optimal in both cases.

After the selection of the optimal parameters (i.e., ntree and mtry), we reran the training-test process by using the best mtry (54 and 94 features) and ntree value (1100 trees), and ten repetitions of tenfold CV with and without the SMOTE exact method, respectively. Table 4 displays the performance metrics of the optimal classifiers. Cohen’s κ values indicate that the optimal classifiers reached a ‘moderate’ degree of inter-rater agreement (Landis & Koch, 1977). It also implies that the application of the SMOTE exact method can improve the overall inter-rater agreement of the classifier. However, the macro and weighted F_1_ scores suggest that the SMOTE exact method did not obviously improve the overall classification performance as the impact from the minority class, the Resolution phase, was still severe.

**Table 4:** The performance metrics of the optimal RF classifiers

Classifiers	Accuracy (SD)	Cohen’s κ (SD)	Macro F_1_ (SD)	Weighed F_1_ (SD)	ntree	mtry
Classifier with the SMOTE exact method	0.730 (0.046)	**0.542 (0.071)**	**0.509 (0.069)**	0.742 (0.056)	1100	54
Classifier without the SMOTE exact method	**0.736 (0.032)**	0.516 (0.063)	0.472 (0.054)	**0.771 (0.061)**	1100	94

The bold values denote the better-performing metrics of the classifier in each row

Tables 5 and 6 illustrate the confusion matrices of the test data in the best cases with and without using the SMOTE exact method. The bold numbers in the diagonal denote the messages that were predicted correctly by the classifiers into five phases of cognitive presence. The error rates indicate that our classifiers had the best performance on the Exploration phase, which accounted for the largest proportion of instances in the data set (see Table 2). The lowest performance was reflected in the Other and Resolution phase, as they had the fewest instances. Our classifiers with the SMOTE exact method obtained a better accuracy for Integration than those without. However, the prediction accuracy for the classes with the fewest instances was still low regardless of using the SMOTE method.

**Table 5:** Confusion matrix of the best classifier with the SMOTE exact method

Predicted labels	Manual labels				
	Other	Triggering	Exploration	Integration	Resolution
Other	**1**	2	1	0	0
Triggering	7	**19**	3	0	0
Exploration	1	7	**74**	8	1
Integration	0	0	6	**13**	1
Resolution	0	0	0	3	**1**
Error rate	0.889	0.321	0.119	0.458	0.667

The bold values denote the number of correct predictions in contrast to the incorrect predictions in the other cells

**Table 6:** Confusion matrix of the best classifier without the SMOTE exact method

Predicted labels	Manual labels				
	Other	Triggering	Exploration	Integration	Resolution
Other	**2**	0	1	0	0
Triggering	4	**21**	3	0	0
Exploration	3	7	**78**	16	2
Integration	0	0	2	**8**	1
Resolution	0	0	0	0	**0**
Error rate	0.778	0.250	0.071	0.667	1.000

The bold values denote the number of correct predictions in contrast to the incorrect predictions in the other cells

### Feature importance analysis—RQ2

**Fig. 1:**
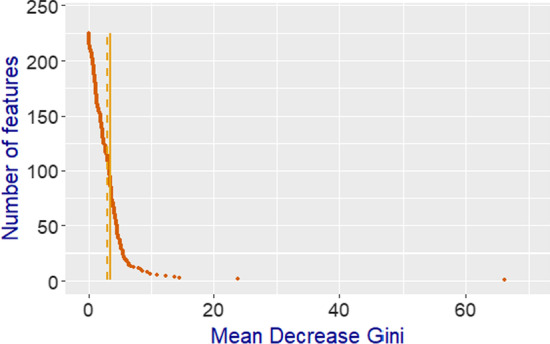
Feature importance by Mean Decrease Gini (MDG) measure with the SMOTE exact method. The solid line displays the average MDG score (3.45), while the dashed line represents the median MDG score (2.97)

We also analysed the importance of the classification features for cognitive presence. Figure 1 demonstrates the importance measures (MDG scores) for all the 225 features used in our classifiers. The scores came from our optimal classifiers with the SMOTE exact method, which obtained higher inter-rater agreement across the skewed data. Most of the features had low MDG scores, whereas only a few features had high scores. We found that most of the classification features were weak indicators for cognitive presence.

Table 8 in the Appendix displays the top 10% of all the classification features ranked by their MDG scores. It also shows the importance scores of each feature to identify every cognitive presence phase separately. In other words, they measure how much including a feature increases the classification accuracy. The mean and SD values of the features for each cognitive phase were also listed for reference.

The linguistics features accounted for the majority of the top 10% features (i.e., 19 of 23). Most of them came from the Coh-Metrix features, and merely two features from the LIWC. The most relevant two features were the number of words in a message (cm.DESWC, first), and the average number of words in each sentence (cm.DESSL second). Their importance scores indicate that the longer messages had a stronger association with the higher phases (i.e., Integration and Resolution). The messages classified into the Triggering event phase had a high probability of using fewer words in the entire message and sentences. The messages classified into the Resolution phase tended to have longer sentences. Apart from the word and sentence lengths features, the strong indicators for the message in the Other were the lower average scores of LSA similarity between verbs (cm.SMCAUSIsa, sixth), and the higher text readability scores for second-language readers (cm.RDL2, 19th). It also reflected a strong association with the Triggering event phase when the messages tended to include more first-person singular pronouns (i.e., ‘I’), children words, non-repeated words, and abstract words (i.e., in contrast to concrete words), according to the results of cm.WRDPRP1s (7th), cm.WRDAOAc (8th), cm.LDTTRa (9th), cm.WRDCNCc (13th), respectively. The messages in the Exploration phase tended to use more concrete words (cm.WRDCNCc, No.13), meaningful content words (cm.WRDMEAc, 18th), specific words (cm.WRDHYPn, 15th) and nouns (cm.WRDNOUN, 23rd). In the messages of higher cognitive phases (i.e., Integration and Resolution), the lexical diversity of words used (cm.LDMTLD, fourth) was higher than the lower phases. This positive relevance was stronger in the messages of the Integration phase. Moreover, the result of an LIWC feature (liwc.cogproc, 16th) implies that higher cognitive phases tended to use more words in the LIWC vocabularies that reflect cognitive processes, and this phenomenon had the strongest association with the messages in the *Exploration* phase. The scores of another LIWC feature (liwc.ipron, 17th) strongly indicate that the messages in the Integration phase tended to use more impersonal pronouns (i.e., ‘it’).

The measures of semantic similarities between the message and its previous and next message are important to identify the cognitive presence phases, according to the scores of sim.cos.pre (3rd), sim.bert.pre (14th), and sim.bert.next (21st) features in Table 8. The messages that had lower semantic similarity (TF-IDF) with their previous messages can be a strong indicator for the Triggering event phase. The message depth (mes.depth, 10th), one of the discussion context features, is also an important indicator. Based on the mean and SD scores, the Resolution phase tended to appear more often at deeper positions of a conversation, whereas messages in the middle of a conversation had a high probability of being in the Exploration phase. Other contextual features, such as whether the message was the start or the end of a conversation, may have low relevance to the classification of cognitive presence in the target MOOC discussions.

Summary of important features for cognitive presence phases in the philosophy MOOC discussions Messages in the

- The other: fewer words, less similar verbs, more readable for second-language readers.
- Triggering event: more ‘I’, children’s words, non-repeated words, abstract words, and verbs, less similar to the previous message.
- Exploration: more nouns, more concrete and specific words, more cognitive processing relevant words, more often in the middle of a conversation.
- Integration: more lexically diverse words, more ‘it’.
- Resolution: more words, more often at a deeper position of a conversation.

### Cross-domain validation of our classifier—RQ3

We also validated the optimal classifier, which was trained by the Philosophy MOOC set, on the sample data of the other three MOOC data. Table 7 displays the inter-rater agreements between the predicted phases by our RF classifier and the manual labels of cognitive presence phases by the two coders. Samples in Table 7 only contained the messages that were classified into the same phase by both coders. The overall agreement between the automatic and manual labels was a percentage agreement of 49.0%, and Cohen’s κ of 0.224 (294 messages). Across the three subsets (disciplines), the sample from the Education MOOC had the highest percentage agreement of 57.7% and Cohen’s κ of 0.371 (97 messages). Samples from Medicine and Humanities achieved the percentage agreement lower than 50%, and Cohen’s κ lower than 0.2.

**Table 7:** The results of inter-rater agreement between the predicted phases by our classifier and manual classification phases by the two expert coders

Disciplines	Messages	% Agreement	Cohen’s κ
Medicine	99	47.5	0.195
Education	97	**57.7**	**0.371**
Humanities	98	41.8	0.158
All	294	49.0	0.241

The bold values denote that the better-performing metrics of our classifiers were achieved on the Education data set

## Discussion

In this section, we analyse the results reported in the previous section to answer each research question individually and discuss them with the relevant literature.

### Model evaluation when training and testing on the philosophy MOOC data—RQ1

Our classifier reached an accuracy of 73.0% and Cohen’s κ of 0.542 at the best case, demonstrating the inter-rater agreement at a moderate level (Landis & Koch, 1977). It answers our first research question that the random forest classifier trained on the Philosophy MOOC data set with the revised classification features achieved better performance than the start-of-the-art classifiers trained by messages from small-scale courses taught in English (Farrow et al., 2019, 2020), and performed slightly lower than the experiments on the small-scale courses taught in Portuguese (Neto et al., 2021). The higher performance in the Portuguese courses compared to the studies on the small-scale courses in English may be because we applied an adapted classification rubric (Hu et al., 2020, 2021) of cognitive phases for the same MOOC, and only used the messages that were classified into the same phase by all the three coders, which could be a more robust training data set. The slightly lower performance in the English courses compared to the Portuguese courses might be due to the differences in languages, as most of the classification features used to train the classifiers are linguistics features. The error rates (in Tables 5 and  6) for predicting the Other and Resolution phase were still high, which aligns with all the previous studies regardless of using the class rebalancing methods or languages (Barbosa et al., 2020; Farrow et al., 2020; Kovanović et al., 2016; Neto et al., 2018, 2021). There were two possible reasons: (1) very few instances of messages were classified in these two phases, and (2) the classification features that we used have limitations to identify them from their adjacent phases (e.g., the Other from Triggering event, and Resolution from Integration). The confusion matrices (Tables 5 and  6) suggest that most of the errors by our classifiers appeared at the adjacent phases of cognitive presence, which is in line with the finding of the manual classification study by the expert coders (Hu et al., 2020, 2021). Therefore, we envisage that finer categorisation (e.g., including additional categories) of cognitive presence is needed to analyse MOOC discussions.

### Feature importance analysis—RQ2

A constructive finding for answering the second research question is the important classification features we used to identify each cognitive phase. We found that the longer messages with higher lexical diversity, which also occur later in a thread, indicate higher phases of cognitive presence. This point is consistent with the findings in the discussions of the small-scale courses (Farrow et al., 2019, 2020; Kovanović et al., 2016). Another finding is that the semantic similarities between the current message and its previous or next message were also important for identifying cognitive presence in the settings of both MOOCs and the small-scale university courses. The semantic similarity represented by the BERT model (Devlin et al., 2019) suggests promising effects on identifying cognitive presence in MOOC discussions compared to the TF-IDF representations. In other words, using the BERT representations can potentially improve the performance of the cognitive classifiers in future studies. The distinct finding is that the classification features, including (1) whether a message is at the start or end of a thread, (2) name-entity words, and (3) the number of questions marks, were low relevance indicators to identify cognitive presence in the MOOC discussion messages. These features differ from what have been found in the small-scale courses (Barbosa et al., 2020; Farrow et al., 2020; Kovanović et al., 2016; Neto et al., 2018, 2021). For instance, the first post in a thread of the Philosophy MOOC discussions has a high probability of reflecting the higher phases of cognitive presence (e.g., Integration and Resolution), whereas the first position of a thread in the small-scale courses was a very strong indicator for a Triggering event (Farrow et al., 2020). The difference in the importance of this feature implies that the MOOC learners might frequently use the posts to record personal reflective thoughts on the course contents rather than asking for help from the instructors or peers. Another point is that the number of question marks indicates a very weak association with predicting any phases of cognitive presence in the MOOC discussions. In contrast, it was revealed as a very strong indicator to identify a Triggering event in all the previous studies on small-scale courses (Farrow et al., 2020; Kovanović et al., 2016; Neto et al., 2021). The difference suggests that the MOOC learners might often use the sentences ending with question marks to deliver their opinions in the discussion messages, such as the case of rhetorical questions, which is similar to the findings in Hu et al.’s (2021) manual classification work of cognitive presence in MOOC discussions. In addition, the teaching content in the Philosophy MOOC contained fewer name-entity words than the courses from other disciplines (e.g., Software engineering and Statistics courses), which often discuss numbers or technical issues. The difference in the importance of the name-entity words feature indicates that identifying cognitive presence is highly domain-relevant and subjective. In Rourke and Anderson’s (2004) and Park (2009)’s studies, they also noted that manual categorisation of cognitive presence is a subjective operation relying on expert coders. Moreover, one of the linguistic features, cognitive processes from the LIWC, indicates a strong association with identifying higher cognitive phases in the MOOC discussions. This finding can be connected with Moore et al.'s (2019, 2020) studies that implied strongly positive associations between the word count of cognitive processes and the forum engagement in both the self- and instructor-paced MOOCs. These findings regarding the important classification features can provide the researchers with hints on the feature extraction and selection for further improvement of the cognitive classifiers for MOOC discussions.

### Cross-domain validation of our classifier—RQ3

We also investigate the potential generalisability of our classifier when applied to the other three disciplines. The results suggest that the overall agreement between the manual classification and model prediction of cognitive phases fell into the ‘fair-level’ (Landis & Koch, 1977). Our classifier performed slightly better on the Education course than on the other two MOOCs. Therefore, the answer to research question 3 was that our classifier trained by the Philosophy MOOC data could not be applied to the MOOCs from the other three disciplines (i.e., Medicine, Education, and Humanities) with sufficient accuracy. The classification performance was better on the Education MOOC. This result is aligned with the finding in Neto et al.’s (2021) work on the small-scale course taught in Portugese, that the classifiers for cognitive presence developed for one discipline was not sufficiently generic to use in the others. Three possible reasons for this could be: Firstly, the different vocabularies and collocations used in each discipline may confuse the machine learning algorithm since most of the classification features that we used were linguistic features; Secondly, the diversity of the pedagogical design and structures of each MOOC may impact the content that learners posted in the online forums (e.g., learners would post their answers to the questions in the MOOC videos or articles, or would propose their questions or thoughts spontaneously. The former would be strongly guided by the course design, whereas the latter may be more unexpected.); Finally, the instructors’ or mentors’ participation in the discussion forums could impact the identification of cognitive presence since it may change the contextual structures of the cognitive phases in each thread. The contextual features such as message depth and the semantic similarities between the adjacent messages were strong indicators for identifying cognitive presence, which have been found in this study and previous studies. Notably, the three presences of the CoI are ‘interdependent’, not isolated (Akyol & Garrison, 2011), and the students’ critical discourse may be impoverished because of the instructors’ absence (Finegold & Cooke, 2006). Thus, the absence of the instructor’s participation in the training data (i.e., the Philosophy MOOC) may lead to confusion for the automatic models when validating it on the sample data with the instructor’s participation (i.e., the Stanford MOOC sets). Future research is needed to enlarge the training data set by including diverse disciplines, different course designs, and self- and instructor-paced courses for seeking higher performance, generalisability and transferability of automatic models to identify cognitive presence. These improvements can significantly contribute to the practical applications of the automatic models to support teaching and learning in future MOOCs.

### Limitations

We acknowledge the limitations of the data sets used in this study. The limited size of the sample data and their unbalanced classes could influence the accuracy and reliability of the automatic classifiers. Although the training data size is similar to those reported in the literature, it is still far below the size required for practical use. Another limitation is that the MOOC platform (i.e., FutureLearn) of the Philosophy course where we collected training data differs from the MOOC platform (i.e., Stanford University open online courses) where we validated the cross-domain application. The different platform designs may affect the distribution of cognitive phases, which also impact the performance of automatic classifiers. The training data needs two or more coders to reliably classify the discussion messages into phases of cognitive presence, which is very time- and labour-consuming. Using a bigger data set from a broader set of MOOC contexts was beyond the scope of this doctoral research. Still, this research provides an initial step towards providing educators, learners, and researchers with robust models to automatically analyse cognitive presence. This research also sheds light on potentially new understanding of the indicators of cognitive presence in MOOC discussion messages, albeit based on the limited data set. This provides a basis for larger future studies to verify such indicators and make such analyses more practically feasible in the future.

## Conclusions

This study makes three main contributions. First, we adapted and applied the state-of-the-art approach created for analysing discussion messages in traditional, small-scale, for-credit courses to automatically identify the phases of cognitive presence on a Philosophy MOOC. The classifier performed a moderate-level agreement, which was slightly better than previous studies. Secondly, the importance of the classification features to identify cognitive presence in MOOC discussions was analysed in depth and compared to those found in traditional, small-scale, for-credit university courses. Finally, we found that cognitive presence classifiers trained on one discipline MOOC data cannot yet be applied to other disciplines with sufficient accuracy. Future research can build on the implications of our findings to develop higher-performing and more generalised classifiers of cognitive presence. These could be practically implemented on MOOC platforms with a diverse range of discipline-based courses to support teaching and learning in real time.

We recommend that to improve the automatic classification performance and to support educators to better detect and diagnose the phases of cognitive presence in MOOCs, future research could (1) use larger data sets including the messages from different MOOC platforms and disciplines for model training and validation, (2) upgrade the automatic classifiers by weighting the classification features with high importance and excluding those with very low importance, (3) consider different MOOC contexts as classification features, such as the pedagogical designs, self- or instructor-paced learning, course objectives, and learners’ demographics and motivations, to build the classifier.

## Figures and Tables

**Table 8:**
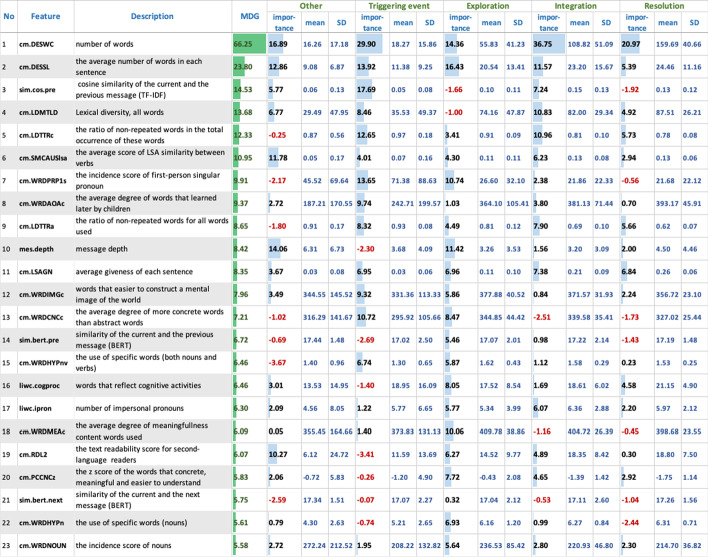
The importance scores of classification features (top 10%) sorted by MDG and for different phases of cognitive presence

## Data Availability

The Philosophy MOOC data used and/or analysed in the study cannot be shared openly due to the ethical issues. The Stanford MOOC datasets used and/or analysed in the study are available from the corresponding author on reasonable request.

## Appendix

See Tables 8, 9 and 10.

**Table 9:** Definitions with message instances of the five categories in cognitive presence

ID	Cognitive phases	Brief definition	Message examples
1	Triggering event	Messages state users’ confusions	“I do find it difficult to override over 30 years of the normalisation of poorly constructed sentences”
2	Exploration	Messages provide information about the cause of the confusion but without a coherent conclusion	“Both overthinking and underthinking leads you to live in low levels of consciousness. I think that [one of the users] explains very well how to find the spot between the two approaches”
3	Integration	Messages propose coherent conclusions to improve the confusing situation with sufficient substantiation	“I think this counter argument works colloquially but not technically it doesn’t follow from the premises that having a job will stop you wanting an iPhone unless you add an implied premise to that effect”
4	Resolution	Messages apply, test, or argue the previous conclusions, usually as new constructs	“Another way to test it would be to see if similar positions eg heads of industry are also held by more left-handed people than statistics would suggest. It would be incredibly difficult to iron out other possible factors…”
0	Other	Messages that do not fall into any of the above categories	“Thanks. I start that Mooc in May”

**Table 10:** Summary of the 225 classification features used in our random forest classifiers

Category	Feature name	Feature description	Quantity
Discussion contextual features	mes.depth	The numeric position (chronological order) within a thread	1
	mes.replies	The total number of replies beneath each message in a thread	1
	mes.start	A binary number to indicate whether the message is the start of a thread	1
	mes.end	A binary number to indicate whether the message is the end of a thread	1
Linguistic features	cm*	Cohesion measure features from the Coh-Metrix tool	108
	liwc*	Word-collection based features from the LIWC tool	90
Semantic similarity	sim.cos.pre	cosine similarity of the current and the previous message represented by two TF-IDF weighted vectors	1
	sim.cos.next	cosine similarity of the current and the next message represented by two TF-IDF weighted vectors	1
	sim.bert.pre	similarity of the current and the previous message represented by pre-trained BERT embedding vectors	1
	sim.bert.next	similarity of the current and the next message represented by pre-trained BERT embedding vectors	1
Name entities	ner*	In each message, occurrence times of 18 types of name entities, including Person, ORG, Date, GPE, Location, Time, etc	18
	ner.total	The total number of all above name-entity types in a message	1

Both Coh-Metrix (3.0 version) and LIWC (2015 version) provided three duplicate features, which were (1) the number of the words in the message, (2) the average number of words in the message, and (3) the number of first-person singular pronouns in the message. Hence, we adopted 198 computational linguistic features, after removing the three duplicate features in LIWC, to build our automatic classifier

## Abbreviations

ANN: Artificial neural network
BERT: Bidirectional encoder representations from transformers
CoI: Community of inquiry
CV: Cross validation
LCT: Logical and critical thinking
LIWC: Linguistic inquiry word Count
LSA: Latent semantic analysis
TF-IDF: Term frequency-inverse document frequency
MDG: Mean decrease gini
MOOC: Massive open online course
RF: Random forest
SD: Standard deviations
SMOTE: Synthetic minority over-sampling technique
